# Associations among *Sebox* and Other MEGs and Its Effects on Early Embryogenesis

**DOI:** 10.1371/journal.pone.0115050

**Published:** 2015-02-13

**Authors:** Min-Woo Park, Kyeoung-Hwa Kim, Eun-Young Kim, Su-Yeon Lee, Jung-Jae Ko, Kyung-Ah Lee

**Affiliations:** Department of Biomedical Science, College of Life Science, CHA University, Pangyo-Ro 335, Bundang-gu, Seongnam-si, Gyeonggi-do, 463–400, Korea; Institute of Zoology, Chinese Academy of Sciences, CHINA

## Abstract

In a previous report, we identified *Sebox* as a new candidate maternal effect gene that is essential for embryonic development and primarily impacts the two-cell (2C) stage. The present study was conducted to determine the mechanism of action for *Sebox* in this capacity, as shown by changes in the expression levels of other known MEG mRNAs after *Sebox* RNA interference (RNAi) in oocytes. *Sebox*-knockdown metaphase II (Mll) oocytes displayed normal morphology, but among the 23 MEGs monitored, 8 genes were upregulated, and 15 genes were unchanged. We hypothesized that the perturbed gene expression of these MEGs may cause the arrest of embryo development at the 2C stage and examined the expression of several marker genes for the degradation of maternal factors and zygotic genome activation. We found that some maternal mRNAs, *c-mos, Gbx2*, and *Gdf9*, were not fully degraded in *Sebox*-knockdown 2C embryos, and that several zygotic genome activation markers, *Mt1a, Rpl23, Ube2a* and *Wee1*, were not fully expressed in conjunction with diminished embryonic transcriptional activity. In addition, *Sebox* may be involved in the formation of the subcortical maternal complex through its regulation of the upstream regulator, *Figla*. Therefore, we concluded that *Sebox* is important in preparing oocytes for embryonic development by orchestrating the expression of other important MEGs.

## Introduction

During fertilization, oocytes resume their meiotic division upon penetration by sperm. Thereafter, the initial cleavage of the zygote early in embryogenesis proceeds without differentiation and growth of the zygote until successful implantation in the mother’s uterus occurs. The particular events that occur during the journey from the oviduct to the uterus rely on factors that are encoded by maternal effect genes (MEGs), which accumulate over the course of oogenesis [1]. A milestone in early embryogenesis that is essential for further embryonic development is the maternal-to-zygotic transition (MZT) [2]. This is the point at which oocyte-specific maternal factors selectively disappear and male or female zygotic genomes are selectively activated. Zygotic genome activation (ZGA) in mice occurs at the two-cell (2C) to four-cell (4C) embryonic transition [3], whereas in bovine, ovine, and human species, this transition occurs at the 4C to eight-cell (8C) stage [4]. Thus, MZT abnormalities may culminate in embryonic arrest or lead to deficiencies in factors that are required for further developmental stages.

Growing oocytes synthesize and accumulate RNAs and proteins that contribute to the normal early embryonic development. Using annealing control primer PCR (described elsewhere), we previously detected differential gene expression levels in the germinal vesicle (GV) and metaphase II (MII) stages of oocyte maturation [5]. We also previously identified that *Sebox* expression was greater in GV than in MII oocytes and that *Sebox* plays a role as an MEG that is essential for embryonic development, functioning primarily at the 2C stage; however, the precise molecular mechanisms of *Sebox* as an MEG have yet to be clarified [6].

**Fig 1:**
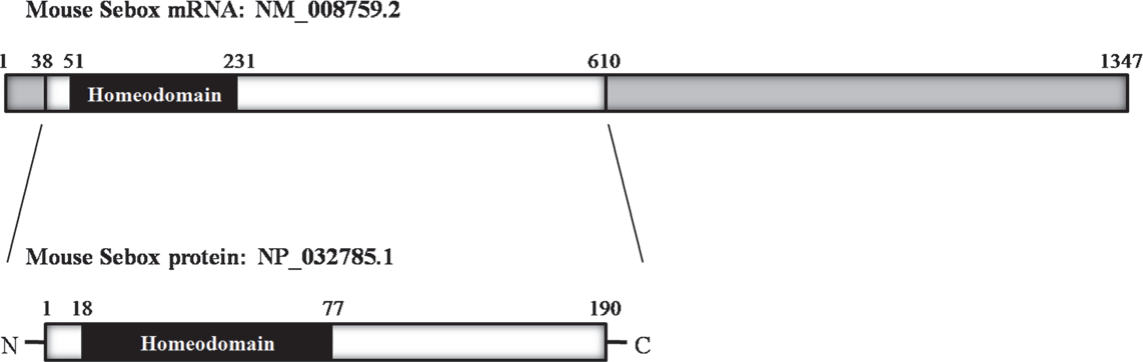
Schematic diagram of the mouse *Sebox* mRNA (NM_008759.2) and protein (NP_032785.1). SEBOX protein has a single homeodomain (black box) near the N-terminus and is considered to be a transcription factor. N, N-terminus; C, C-terminus.

Recently, other sources have substantiated the importance of *Sebox* in early oogenesis [7]. *Sebox* is a mouse paired-like homeobox gene that encodes a transcription factor with a 60 amino acid single homeodomain motif (Fig. 1). In 2000, Cinquanta and colleagues reported the Sebox expression in skin, brain, oocytes, and 2-cell stage embryos [8]. Homeobox genes are a large class of transcriptional regulators that are essential for regulating cell differentiation and the formation of body structures during early embryonic development. Homeobox genes share a highly conserved DNA-binding domain of 60 amino acids, named the homeodomain, which binds to a specific DNA sequence and regulates expression of genes. Therefore, proteins that include a homeodomain play an essential role in both intracellular interactions and control of the expression of target genes.

MEGs were first described in *Drosophila* [9], but the concept of mammalian MEGs was first reported in 2000 [10], with the subsequent discovery of approximately 30 MEGs. MEGs are generally grouped by function during embryonic development as follows: 1) degradation of maternal factors, 2) chromatin remodeling, 3) transcriptional activity, 4) DNA methylation, 5) subcortical maternal complex (SCMC), and 6) pre-implantation development [11]. Therefore, due to their major role in embryogenesis, mutations of MEGs not only place embryonic development in jeopardy but may also compromise oocyte maturation and meiotic division. The present study was conducted to explore the role(s) of *Sebox* in early embryogenesis, assessing the influence of the loss-of-function of *Sebox* on the expression levels of other MEGs in oocytes and on early embryogenesis, particularly the degradation of maternal factors and the transcriptional activity of zygotes during MZT.

## Materials and Methods

### Research animals

ICR mice (female and male), exclusively provided by Koatech (Pyeoungtack, Korea), were mated to produce embryos in the breeding facility at the CHA Research Institute of CHA University. All procedures described herein were reviewed and approved by the Institutional Animal Care and Use Committee of CHA University and were performed in accordance with Guiding Principles for the Care and Use of Laboratory Animals.

### Isolation of oocytes and embryos

Three-week-old female ICR mice were injected with 5 IU pregnant mare’s serum gonadotropin (PMSG; Sigma-Aldrich, St. Louis, MO, USA) and sacrificed 46 h later. Cumulus-enclosed oocyte complexes were then recovered from the ovaries by puncturing preovulatory follicles with 27-gauge needles. M2 medium (Sigma-Aldrich) containing 0.2 mM 3-isobutyl-1-methyl-xanthine (IBMX; Sigma-Aldrich) was used to inhibit germinal vesicle breakdown (GVBD). Cumulus cells were mechanically retrieved from oocytes by repeated extraction through a fine-bore pipette. Isolated murine oocytes were snap frozen and stored at -70°C prior to RNA isolation. Other female mice were superovulated and mated, and pronuclear embryos (PNs) were obtained 18–20 h after hCG injection.

### Messenger RNA isolation

mRNA was isolated from oocytes and embryos at differing developmental stages using the Dynabeads mRNA DIRECT kit (Dynal Asa, Oslo, Norway) according to the manufacturer’s instructions. In short, oocytes were resuspended in 300 μl lysis/binding buffer (100 mM Tris-HCl [pH 7.5], 500 mM LiCl, 10 mM EDTA, 1% LiDS, and 5 mM dithiothreitol [DTT]) for 5 min at room temperature. After vortexing, 20 μl prewashed Dynabeads oligo dT_25_ was mixed with the lysate and annealed by rotating 5 min at room temperature. The beads were separated with a Dynal MPC-S magnetic particle concentrator, and poly(A)^+^ RNAs were eluted by incubation in 14 μl Tris-HCl (10 mM Tris-HCl, pH 7.5) at 73°C for 2 min.

### Reverse-transcriptase polymerase chain reaction (RT-PCR)

Purified mRNA and 0.5 μg oligo (dT) primer were mixed and incubated at 70°C for 10 min, and cDNA was synthesized. Single oocyte- and single embryo-equivalent cDNAs were used as templates for PCR analysis. Primer sequences for the genes encoding *Sebox*, *Figla* and *H1foo* and PCR conditions are listed in Table 1. Thereafter, PCR products were separated by 1.5% agarose gel electrophoresis and analyzed using the Gel Doc EZ Imager (Bio-Rad). Relative gene expression levels were normalized to those of *H1foo*. All experiments were repeated three times.

**Table 1:** Primer sequences and Real-time RT-PCR conditions.

Gene	Accession numbers	Primer sequence*	Annealing temperature	Product size
*Sebox-A*	NM_008759	F-AAAGCCAGGAGCCCTAAACT	60°C	334 bp
		R-TTAGAAGTGGTCTACATTGG		
*Sebox-B*	NM_008759	F-GGAACATCAAGCCATCCTCT	60°C	293 bp
		R-GGCCAGAGCCAAGACTTAAA		
*GFP*	KF111246.1	F-CTGAAGTTCATCTGCACCAC	60°C	334 bp
		R-CGGCCATGATATAGACGTTG		
*H1foo*	NM_138311	F-AAGGAAGATGGCAGACATGG	60°C	137 bp
		R-TCTTTGCCTTCCTGACCCTA		
*Actb*	NM_007393.3	F-GGGTGTGATGGTGGGAATGGG	60°C	489 bp
		R-GCTGTGGTGGTGAAGCTGTAG		
*Gapdh*	BC092294	F-ACCACAGTCCATGCCATCAC	60°C	451 bp
		R-TCCACCACCCTGTTGCTGTA		
*Ago2*	NM_153178.4	F-AGAACATGACAGCGCTGAAG	60°C	115 bp
		R-AAAGTACATGGTGCGCAGTG		
*Atg5*	NM_053069.5	F-GCCTTTCATCCAGAAGCTG	60°C	149 bp
		R-TTGGCTCTATCCCGTGAATC		
*Bmp15*	NM_009757.4	F-TACAAGGTCAGCTTCCACCA	60°C	135 bp
		R-ATGGCATGGTTGGGTGAA		
*Bnc1*	NM_007562.2	F-ACCATCCTGGATTTGAGCAC	60°C	118 bp
		R-TGCCATCACTGTCCTCCATA		
*Brg1*	NM_001174078.1	F-CGGCAGAAGATTGAGAAGGA	60°C	119 bp
		R-CCCAGCTTGATCTTCACCTT		
*Btg1*	NM_007569	F-CGACAGCTGCAGACTTTCAG	60°C	238 bp
		R-GGTAGGACACTTCGTAGGGG		
*Cdc2*	NM_007659.3	F-GGACTACAAGAACACCTTTC	60°C	262 bp
		R-CAGGAAGAGAGCCAACGGTA		
*c-mos*	NM_020021	F-TGGCTGTTCCTACTCATTTC	60°C	273 bp
		R-CTTTATACACCGAGCCAAAC		
*Dicer1*	NM_148948.2	F-AGTCTCTTGCTGGTGCCATT	60°C	148 bp
		R-GGTTCCATCTCGAGCAATTC		
*Dnmt3a*	NM_007872.4	F-CCCTTCTTCTGGCTCTTTGA	60°C	117 bp
		R-TGCAGCAGACACTTCTTTGG		
*Dnmt3l*	NM_019448.3	F-TGCTGACTGAGGATGACCAA	60°C	103 bp
		R-ACCCGCATAGCATTCTGGTA		
*Eif1a*	NM_010120.5	F-ATGCTGGGAAATGGACGGTT	60°C	196 bp
		R-AGGCCTTCAGACTTCTTGCT		
*Figla*	NM_012013.1	F-TGTTCTGGAAGAAGCGAAGG	60°C	117 bp
		R-TGGGTAGCATTTCCCAAGAG		
*Filia*	NM_025890.3	F-ATGGAGAGCACATCCCACA	60°C	148 bp
		R-TGAGCCAGATCAGTGAGCA		
*Floped*	NM_026480.3	F-ATCTTTGGACAACCCAGTGC	60°C	144 bp
		R-TAGGATTGAGGAGGCACGAA		
*Gbx2*	NM_010262.3	F-ATTTGCCTGGTCAGACTGCT	60°C	363 bp
		R-TGCTAACGTGAACAGGGATG		
*Gdf9*	NM_008110.2	F-TTGGCAGTCTCTTCAGTCCA	60°C	106 bp
		R-GGGAGATCTTTCCACCTCAA		
*Hr6a*	NM_019668.3	F-CCAATAGTCCAGCAAACAGCC	60°C	100 bp
		R-TCGCGCCAGCTTTGTTCTA		
*Hsf1*	NM_008296.2	F-CAACAACATGGCTAGCTTCG	60°C	136 bp
		R-CTGTCCACGCAAGAAACAAG		
*Hsp70.1*	NM_010478.23	F-AACGTGCTCATCTTCGACCT	60°C	185 bp
		R-TGGCTGATGTCCTTCTTGTG		
*Klf4*	NM_010637	F-AAAAGAACAGCCACCCACAC	60°C	227 bp
		R-GAAAAGGCCCTGTCACACTT		
*Kpna1*	NM_008465.5	F-TCCAAGCAGTCATCGATGCA	60°C	250 bp
		R-TGTGCCCTATTTCCAGCTGT		
*Mater*	NM_001039143.1	F-CCTTGGGAATGCCTTGAGTA	60°C	112 bp
		R-GTTGCTGAAAAGGGCTGAGA		
*Mt1a*	NM_001039368.1	F-AAAATCTTTGTGGGCAGCCG	60°C	186 bp
		R-TCTCTGCATCTGATGGGATC		
*Muerv-l*	Y12713	F-TTGCTTCCTGTCCCCATAAC	60°C	132 bp
		R-AAAATGACCAGGGGGAAGTC		
*Nobox*	NM_130869.3	F-TTTCCCATCCCTTCAGTCAC	60°C	123 bp
		R-TCTCCACTGAAGCCAAAAGG		
*Npm2*	NM_181345.3	F-GAAAGCCAAAGAGGAGGTGA	60°C	146 bp
		R-GCCGAAAAGTTACTGGAGGA		
*Oct4*	NM_013633.3	F-CCGGAAGAGAAAGCGAACTA	60°C	112 bp
		R-CTGATTGGCGATGTGAGTGA		
*Omt2b*	NM_205822.2	F-AGCAGACAGAAGGCAGCATT	60°C	215 bp
		R-AGCAATAGTTCCGGCCTCAA		
*Padi6*	NM_153106.2	F-TGGGAGGGAGAGCAAAACTA	60°C	129 bp
		R-TTGTCCTCCAATCCCAGTTC		
*Rpl23*	NM_022891.3	F-CATGGTGATGGCCACAGTTA	60°C	136 bp
		R-GACCCCTGCGTTATCTTCAA		
*Stella*	NM_139218.1	F-TGTTGTCGGTGCTGAAAGAC	60°C	151 bp
		R-CACTGTCCCGTTCAAACTCA		
*Tcl1*	NM_009337.3	F-GAAGCTATGTCCCCCAGTCA	60°C	150 bp
		R-TTCAAGCAACATGTCCTCCA		
*Tif1alpha*	NM_145076.3	F-ACCCAATGGACTTGTCAACC	60°C	148 bp
		R-CCAGCATTGGCTACTTCAGA		
*Tle6*	NM_053254.2	F-AACCTCAAAGGCCCTACCAA	60°C	134 bp
		R-TGGAACAGATGCTCCAGTGA		
*Ube2a*	NM_019668.3	F-AATGGTTTGGAATGCGGTCA	60°C	272 bp
		R-TGTTTGCTGGACTATTGGGA		
*U2afbp-rs*	NC_000077.6	F-TAAGCTGCAACCTGGAACCT	60°C	109 bp
		R-CCTGCGTACCATCTTCCATT		
*Uchl1*	NM_011670.2	F-GCCCAGCATGAAAACTTCAG	60°C	150 bp
		R-CAGCTTGTCTTGGTTGTTGG		
*Wee1*	NM_009516.3	F-AGCCATCTACCGAAAGCAGA	60°C	375 bp
		R-ATCTGTGAAGAGTGCCCGTT		
*Zar1*	NM_174877.3	F-GTTCTGCCGAGTGTGTGAGA	60°C	143 bp
		R-CACACAAGTCTTGCCGATGG		
*Zfp57*	NM_001013745.2	F-CAGCCATCCAGGACACCAG	60°C	144 bp
		R-GCTTCCGACAAATGTCAGGTT		
*Zscan4*	NR_033707.1	F-CAGATGCCAGTAGACACCAC	60°C	514 bp
		R-GTAGATGTTCCTTGACTTGC		

*F = Forward, R = Reverse

### Quantitative real time RT-PCR

Quantitative real time RT-PCR analysis of embryonic MEG mRNA relied on the iCycler iQ Detection System (Bio-Rad Laboratories Inc, Hercules, CA, USA). iQ SYBR Green Supermix PCR reagents (Bio-Rad) were used to monitor amplification, and the results were analyzed using the iCycler iQ proprietary software. The reaction mixture contained cDNA, 20 pmol forward and reverse primers, and SYBR Green Supermix 2 (100 mM KCl, 40 mM Tris-HCl [pH 8.4], 0.4 mM each dNTP, 50 U/ml iTaq DNA polymerase, 6 mM MgCl_2_, SYBR Green I, 20 nM fluorescein, and stabilizers). The primer sequences used for the genes analyzed are listed in Table 1. Templates were amplified through 40 cycles of denaturation (40 sec, 95°C), annealing (40 sec, 60°C), and extension (40 sec, 72°C). Upon completion of PCR, fluorescence was monitored continuously as the samples were slowly heated from 60°C to 95°C at 0.5°C intervals. The melting curves were used to identify any nonspecific amplification products. The expression levels of each mRNA species in oocytes and embryos were normalized to those of *H1foo* and *Actb*, respectively. The relative expression levels of the target genes were evaluated using the comparative C_T_ method [12, 13], and all analytic procedures were repeated at least three times.

### Preparation of *Sebox* and *GFP* dsRNA

*Sebox*-A and *GFP* primers were used to amplify regions of *Sebox* and *GFP* cDNA, respectively, which were then cloned into pGEM-T Easy (Promega, Madison, WI, USA) and linearized with SpeI. A MEGAscript RNAi Kit (Ambion, Austin, TX, USA) and T7 RNA polymerase were used to synthesize single-stranded RNA (ssRNA) for each orientation. Complementary RNAs were mixed and incubated 5 min at 75°C and then cooled to room temperature. The formation of dsRNA was verified by 1% agarose gel electrophoresis, comparing the mobility of dsRNA with that of ssRNA. For microinjection, RNAs were diluted to a final concentration of 2 μg/μl. *GFP* RNAi was used as injection control.

### Microinjection and *in vitro* culture

GV oocytes and PN embryos were microinjected with *Sebox* and *GFP* dsRNA in M2 medium containing 0.2 mM IBMX or in M2 medium alone, respectively. An injection pipette holding dsRNA solution was inserted into the cytoplasm of oocytes or embryos, and 10 pl dsRNA was microinjected with a constant-flow system (Femtojet; Eppendorf, Hamburg, Germany). To achieve the Mll stage, oocytes were cultured in M16 containing 0.2 mM IBMX for 8 h, followed by culture in plain M16 for 16 h in 5% CO_2_ at 37°C. Similarly, *GFP* and *Sebox* dsRNA-microinjected PN embryos were developed to the 2C stage in M16 medium containing 100 μM EDTA (Sigma-Aldrich).

### Transcriptional activity assay

Newly synthesized RNAs, e.g., transcriptional activity, in embryos may be visualized by applying 5-ethynyl uridine (EU) to an *in vitro* embryonic transcriptional activity assay [12]. The Click-iT RNA Imaging Kit (Invitrogen, Carlsbad, CA, USA) was used for this purpose. After subjecting embryos to culture for 1 h in 2 mM EU-supplemented medium, embryos were washed three times for 10 min and fixed in 3.7% formaldehyde for 1 h. The preserved embryos were washed three times for 10 min and permeabilized by exposure to 0.2% Triton X-100 for 10 min. Finally, embryos were sequentially immersed in reaction buffer for 30 min, washed three times, and examined by confocal microscopy after the reaction buffer was eliminated with rinse buffer.

### Statistical analysis

Statistical analysis of real time PCR data was carried out using student’s t-test. Data derived from at least three separate and independent experiments were expressed as the mean ± SEM. The *p* values were calculated based on a paired *t-test* of the triplicate delta C_T_ values for each gene in the *GFP* RNAi group and *Sebox* RNAi group, and a value of *p*<0.05 was considered statistically significant.

## Results

### Expression levels of other MEGs impacted by *Sebox* RNAi

**Fig 2:**
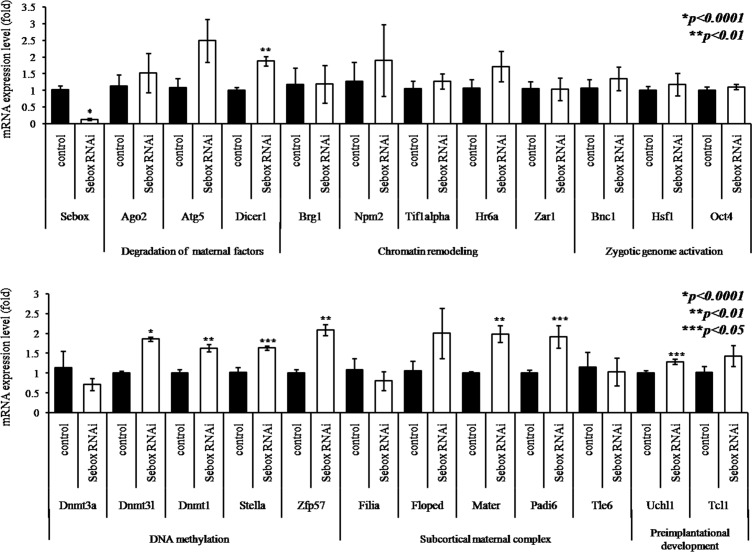
Altered expression of 23 MEGs in *Sebox*-knockdown Mll oocytes. Quantitative real time RT-PCR experiments were repeated at least three times, expressing data as the mean±SEM. Expression levels were calculated from the C_T_ values after normalization with H1foo. The statistical significance was assessed by a paired *t*-test with *p* values obtained by paired *t-test* within the delta C_T_ values. Asterisks, *, **, and ***, represent statistical significance at *p<0*.*0001*, *p<0*.*01* and *p<0*.*05*, respectively. Control, GFP dsRNA-injected MII oocyte; *Sebox* RNAi, *Sebox* dsRNA-injected MII oocyte.

We previously reported the expression of *Sebox* mRNA in GV oocytes [6]. Knockdown of *Sebox* mRNA and protein in GV oocytes did not affect the meiotic cell cycle of oocytes, so the oocytes without *Sebox* expression developed to MII but were arrested at the 2C stage of early embryonic development. Although *Sebox*-knockdown GV oocytes developed to normal MII in appearance, the expression levels of the 8 among 23 studied MEGs were up-regulated (Fig. 2). These results suggest that *Sebox* is an important regulatory transcription factor that may function in controlling the expression of other MEGs during preimplantational embryonic development. In particular, 1 gene pertaining to degradation of maternal factors (*Dicer*), 4 genes related to DNA methylation (*Dnmt3l*, *Dnmt1*, *Stella*, and *Zfp57*), and 2 genes pertaining to SCMC organization (*Mater*, *and Padi6*) were up-regulated after *Sebox* RNAi knockdown (Fig. 2). The expression of *Uchl1* related to preimplantation development was also up-regulated.

### Inadequate selective maternal mRNA degradation in *Sebox* knockdown 2C embryos

**Fig 3:**
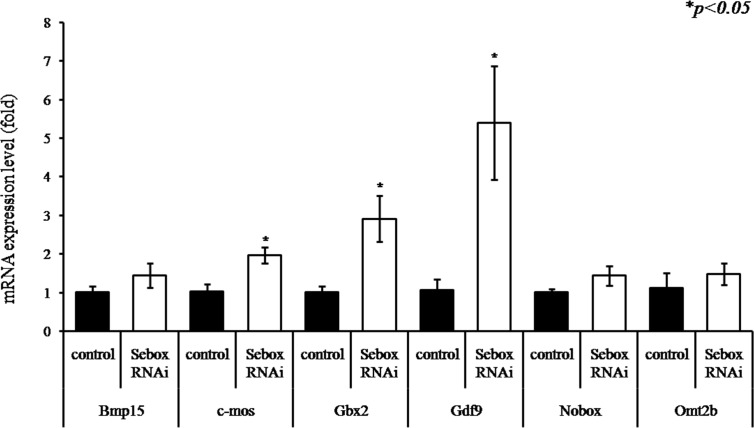
Expression levels of selected maternal mRNAs in *Sebox*-knockdown 2C embryos. To investigate the role of *Sebox* in degrading maternal mRNAs, *Sebox* RNAi knockdown PN embryos were developed to the 2C stage, and expression levels of maternal factors were determined by real time RT-PCR. Maternal factors that are typically absent in 2C control embryos, *c-mos*, *Gbx2*, *Gdf9*, were not degraded in *Sebox*-knockdown embryos arrested at the 2C stage (*p<0.05). The statistical significance was assessed by a paired *t*-test. Control, GFP dsRNA-injected 2C embryo; *Sebox* RNAi, *Sebox* dsRNA-injected 2C embryo.

To determine the exact effects of the up-regulated expression levels of several MEGs on the 2C arrest after *Sebox* RNAi knockdown, particularly on the degradation of some maternal factors, we measured the degradation of several well-known maternal factors. Changes in expression of the maternal mRNAs *Bmp15*, *c-mos*, *Gbx2*, *Gdf9*, *Nobox*, and *Omt2b* were evident in 2C stage embryos after *Sebox* RNAi knockdown (Fig. 3). Compared with control 2C stage embryos, the expression levels of *c-mos*, *Gbx2*, *and Gdf9* were relatively high in arrested 2C embryos after *Sebox* RNAi knockdown. The expression levels of the other known maternal factors, such as *Nobox* and *Omt2b*, were not changed. These results strongly suggest that *Sebox* is fairly involved in the process of degrading maternal factors.

### Incomplete expression of ZGA markers after *Sebox* RNAi knockdown

**Fig 4:**
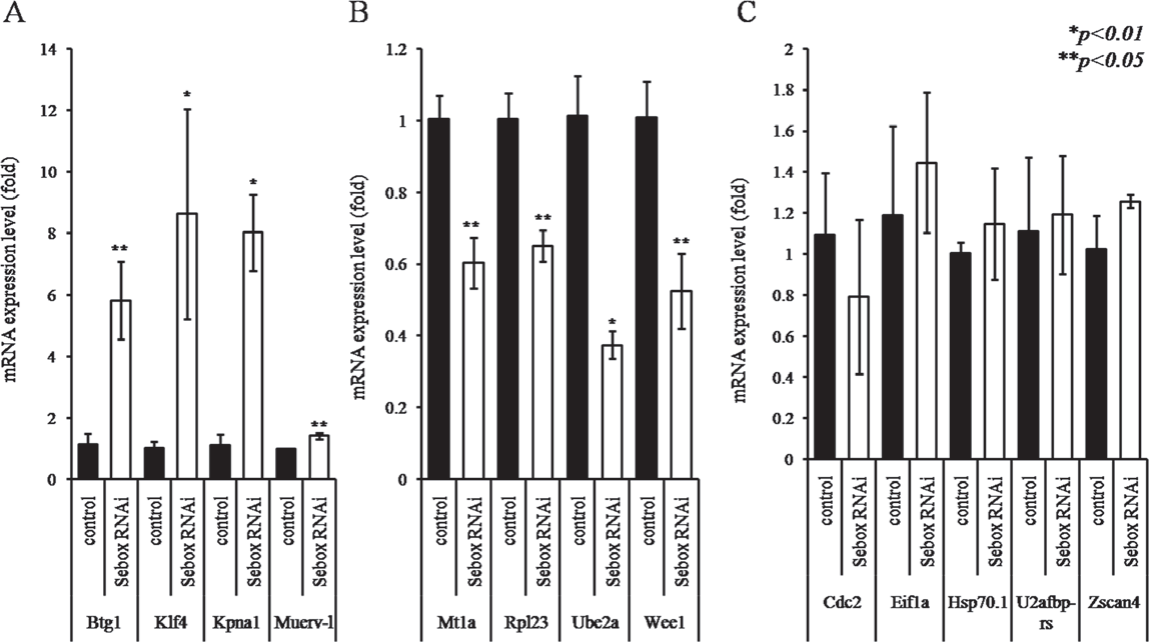
Expression of zygotic genome activation (ZGA) markers in *Sebox*-knockdown 2C embryos. To investigate the role of *Sebox* in ZGA, *Sebox* RNAi knockdown PN embryos were developed to the 2C stage, and the expression levels of 13 marker genes were determined by real time RT-PCR. The expression of 4 genes (*Btg1*, *Klf4*, *Kpan1* and *Muerv-l*) were up-regulated (A), and 4 genes (*Mt1a*, *Rpl23*, *Ube2a* and *Wee1*) were down-regulated in *Sebox*-knockdown 2C embryos (B); while the expression levels of 5 genes (*Cdc2*, *Eif1a*, Hsp70.1, *U2afbp-rs*, and *Zscan4*) were unchanged. Asterisks, * and **, represent statistical significance at *p<0*.*01* and *p<0*.*05*, respectively. Control, GFP dsRNA-injected 2C embryo; *Sebox* RNAi, *Sebox* dsRNA-injected 2C embryo.

Embryos subjected to *Sebox* RNAi knockdown were blocked at the 2C stage of embryonic development. Normal embryonic development requires ZGA, which should be indicated by expression of typical ZGA markers [13,14]. Thus, we determined the expression levels of known ZGA markers by comparing the 2C controls and 2C *Sebox*-knockdown zygotes (Fig. 4A-C). The expression levels of Btg1, Klf4, Kpna1, Muerv-1 were significantly up-retulated, while the expression of *Mt1a*, *Rpl23*, *Ube2a* and *Wee1* were down-regulated after *Sebox* RNAi knockdown. Expression of *Cdc2*, *Eif1a*, *Hsp70*.*1*, *U2afbp-rs* and *Zscan-4* were not significantly changed. These results demonstrate that *Sebox* is partly, but not exclusively, involved in ZGA.

### Diminished transcriptional activity in *Sebox* knockdown 2C embryos

**Fig 5:**
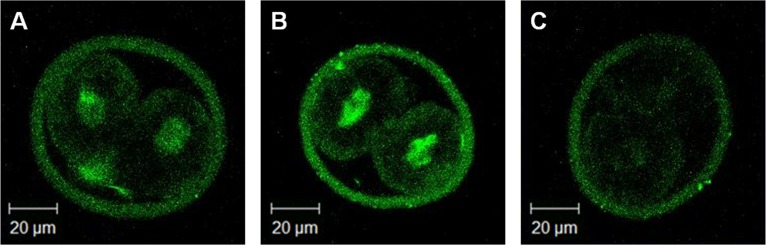
Transcriptional activity assay of *Sebox*-knockdown 2C embryos. Embryonic transcriptional activities were investigated by measuring embryonic nuclear EU incorporation. Control (A) and sham control (B) embryos showed normal levels of nuclear transcriptional activity, whereas transcriptional activity in *Sebox*-knockdown embryos (C) was diminished by comparison.

Due to the imperfect pattern of expression of several ZGA markers described above, we decided to evaluate the transcriptional activity of the 2C embryos by measuring EU incorporation in embryos with or without *Sebox* RNAi knockdown. *Sebox*-knockdown embryos showed dramatically decreased EU incorporation compared with control and sham-control embryos, confirming that the halted maternal factor degradation and ZGA during the MZT period caused by *Sebox* RNAi knockdown resulted in decreased transcription in embryos arrested at the 2C stage (Fig. 5).

### Expression of *Figla* in *Sebox*-knockdown Mll oocytes

**Fig 6:**
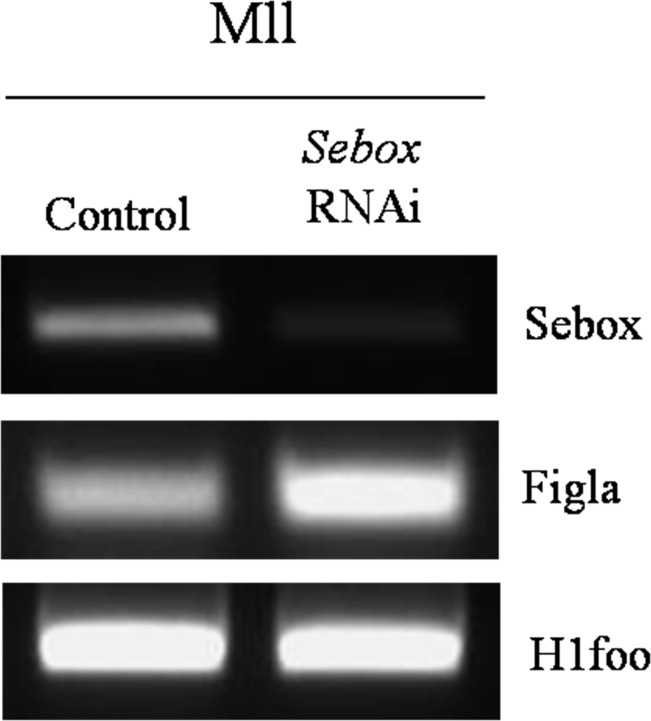
Expression of *Figla* in *Sebox*-knockdown Mll oocytes. Up-regulated expression of *Figla*, a known upstream regulator of SCMC, was confirmed in *Sebox*-knockdown Mll oocytes. Control, GFP dsRNA-injected MII oocyte; *Sebox* RNAi, *Sebox* dsRNA-injected MII oocyte.

Because 2 out of the 5 genes involved in SCMC formation, i.e., *Mater*, and *Padi6*, were up-regulated in *Sebox*-knockdown Mll oocytes, we evaluated changes in the known upstream regulators of SCMC. *Figla*, a germ-cell-specific, basic helix-loop-helix transcription factor, has been reported to be a key regulatory molecule in coordinating the expression of the NALP family of genes [15]. Three members of the NALP gene family have oocyte-specific expression [16]. Among them, *Nalp5* (also known as *Mater)* is important in the formation of the SCMC complex [17]. Thus, we evaluated the expression levels of *Figla* after *Sebox* RNAi knockdown and found that expression levels increased 11.7-fold in *Sebox*-knockdown Mll oocytes compared with the controls (Fig. 6). Consequently, we concluded that *Sebox*, either directly and/or indirectly through *Figla*, regulates the expression of SCMC component genes.

## Discussion

During the MZT, gene expression is dramatically altered as a necessary step in embryonic development. By definition, MEGs are transcribed during oogenesis and are required for early developmental activities, such as establishing the overall polarity of the embryo. Some MEGs are expressed only in female gametes, whereas others are expressed after the embryonic genome is activated [18]. The timing of embryonic gene activation is species-specific [19]. In mice, embryonic gene activation occurs at the 2C stage, concurrently with the degradation of most maternal mRNA transcripts [20]. Global expression profiles have identified distinctive patterns of maternal mRNA degradation and zygotic genome activation in mice, indicating remarkably dynamic reprogramming of gene expression at the 2C stage [21–23].

One major point of inquiry was whether developmental repercussion is found in *Sebox*-knockdown 2C embryos. In this study, *Sebox*-deficient MII oocytes displayed altered expression of several MEGs. First, the role of *Sebox* in degrading maternal factors was investigated. The degradation of maternal factors is initiated during oocyte maturation and proceeds after fertilization [24]. To support early embryogenesis, the degradation of previously existing factors is a crucial and selective process [25]. We measured the expression of known maternal mRNAs (*Bmp15*, *c-mos*, *Gbx2*, *Gdf9*, *Nobox*, and *Omt2b*), all of which should be degraded in normal 2C zygotes, and found incomplete elimination of *c-mos*, *Gbx2*, and *Gdf9* after *Sebox* RNAi knockdown. Such abnormal clearance of maternal factors likely translates to latent defects in embryonic development.

Next, we confirmed the presence of abnormal ZGA and found that *Mt1a*, *Rpl23*, *Ube2a* and *Wee1* were down-regulated after *Sebox* RNAi knockdown but that *Cdc2*, *Eif1a*, *Hsp70*.*1*, *U2afbp-rs*, *and Zscan4* were not. Furthermore, expression of 4 more genes, *Btg1*, *Klf4*, *Kpna1*, and *Muerv-1* were even up-regulated after the loss of *Sebox*. This finding suggests that *Sebox* is certainly a significant regulator of ZGA, but it is not critical or exclusive because the expression levels of 5 out of 13 genes were not affected. SEBOX contains a homeodomain and may thus act as a transcription factor [8,26,27]. Indeed, a transcriptional activity assay confirmed a reduction of embryonic transcriptional activity after *Sebox* RNAi knockdown. Therefore, the regulation of ZGA by SEBOX and its control over the expression of other MEGs may occur at the transcriptional level. Further research on the interrelationship between SEBOX as a transcriptional factor and promoters of altered MEG expression levels is required.

An interesting outcome of this study was the finding that the expression levels of SCMC components were increased after *Sebox* RNAi knockdown. The SCMC encompasses many maternal proteins, of which FILIA, FLOPED, MATER, PADI6, and TLE6 are crucial for progression beyond the first embryonic cell division [17]. Among these components, FLOPED, MATER, and TLE6 proteins show interactivity, whereas Filia and MATER bind directly in embryos [28]. According to previous findings, *Figla* is a key regulatory molecule of *Nalp5*, also known as *Mater* [16], and MATER has an important role in SCMC complex formation [17,28]. We confirmed a relationship between *Sebox* and *Figla* and found it noteworthy that *Sebox* depletion up-regulated *Figla* expression. These findings strongly suggest that *Sebox*, *Figla*, and SCMC components are linked. The specific interrelationships of MEGs have not been fully elucidated. Our results may provide a greater impetus to probe such relationships, exploring the direct/indirect interplay among SEBOX, FIGLA, and other MEGs, at both the transcriptional and post-translational levels.

Other publications have stressed the importance of the MZT in early embryonic development [29–31]. Arrest of α-amanitin-treated embryos at the 1C or 2C stage has been documented [32], and developmental block at the 2C stage has been attributed to delayed ZGA [33]. However, the specific molecular mechanism of the MZT in mice is still unclear. We believe that SEBOX is an important regulator of the MZT in addition to the genes that have been discovered to be active during the MZT [34].

Aside from their impact on embryonic development, a variety of functions have been ascribed to many MEGs in oocytes. *Basonuclin*-deficient oocytes containing cytoplasmic granules have been found to arrest at the 2C stage [35]; *Ctcf*-deficient oocytes showed delayed GVBD and embryonic developmental arrest [36]; and *Padi6* is thought to regulate microtubular and organelle dynamics during oocyte maturation and to contribute to the SCMC during early embryogenesis [37]. We previously reported that *Gas6* contributes to the cytoplasmic maturation of oocytes and PN formation [38]. Additionally, in the present study, we report that even though *Sebox*-knockdown oocytes developed to the MII stage with normal morphology, *Sebox* knockdown may contribute to the incompetent cytoplasmic maturation of oocytes, which affects early embryo development.

In conclusion, our findings support an intimate association between *Sebox* and other MEGs, whereby *Sebox* is involved in regulating the elimination of maternal factors and promotion of embryonic gene expression required for normal developmental progression. These perturbed cytoplasmic expression levels that we observed for various genes in *Sebox*-deficient mouse oocytes signify impaired fertilization and embryonic development and thus merit further investigation.

## References

[1] Latham KE (1999) Mechanisms and control of embryonic genome activation in mammalian embryos. Int Rev Cytol 193: 71–124. 10494621 10.1016/s0074-7696(08)61779-9

[2] Schultz RM (2002) The molecular foundations of the maternal to zygotic transition in the preimplantation embryo. Hum Reprod Update 8: 323–331. 12206467 10.1093/humupd/8.4.323

[3] Wang H, Dey SK (2006) Roadmap to embryo implantation: clues from mouse models. Nat Rev Genet 7: 185–199. 16485018 10.1038/nrg1808

[4] Telford NA, Watson AJ, Schultz GA (1990) Transition from maternal to embryonic control in early mammalian development: a comparison of several species. Mol Reprod Dev 26: 90–100. 2189447 10.1002/mrd.1080260113

[5] Yoon SJ, Chung HM, Cha KY, Kim NH, Lee KA (2005) Identification of differential gene expression in germinal vesicle vs. metaphase II mouse oocytes by using annealing control primers. Fertil Steril 83 Suppl 1: 1293–1296. 15831304 10.1016/j.fertnstert.2004.09.037

[6] Kim KH, Kim EY, Lee KA (2008) SEBOX is essential for early embryogenesis at the two-cell stage in the mouse. Biol Reprod 79: 1192–1201. 10.1095/biolreprod.108.068478 18753614

[7] Moreno DL, Salazar Z, Betancourt M, Casas E, Ducolomb Y, et al (2014) Sebox plays an important role during the early mouse oogenesis in vitro. Zygote 22: 64–68. 10.1017/S0967199412000342 22805237

[8] Cinquanta M, Rovescalli AC, Kozak CA, Nirenberg M (2000) Mouse Sebox homeobox gene expression in skin, brain, oocytes, and two-cell embryos. Proc Natl Acad Sci U S A 97: 8904–8909. 10922053 10.1073/pnas.97.16.8904 PMC16794

[9] Nusslein-Volhard C, Lohs-Schardin M, Sander K, Cremer C (1980) A dorso-ventral shift of embryonic primordia in a new maternal-effect mutant of Drosophila. Nature 283: 474–476. 6766208 10.1038/283474a0

[10] Tong ZB, Gold L, Pfeifer KE, Dorward H, Lee E, et al (2000) Mater, a maternal effect gene required for early embryonic development in mice. Nat Genet 26: 267–268. 11062459 10.1038/81547

[11] Li L, Zheng P, Dean J (2010) Maternal control of early mouse development. Development 137: 859–870. 10.1242/dev.039487 20179092 PMC2834456

[12] Waksmundzka M, Debey P (2001) Electric field-mediated BrUTP uptake by mouse oocytes, eggs, and embryos. Mol Reprod Dev 58: 173–179. 11139229 10.1002/1098-2795(200102)58:2<173::AID-MRD6>3.0.CO;2-2

[13] Zeng F, Schultz RM (2005) RNA transcript profiling during zygotic gene activation in the preimplantation mouse embryo. Dev Biol 283: 40–57. 15975430 10.1016/j.ydbio.2005.03.038

[14] Kigami D, Minami N, Takayama H, Imai H (2003) MuERV-L is one of the earliest transcribed genes in mouse one-cell embryos. Biol Reprod 68: 651–654. 12533431 10.1095/biolreprod.102.007906

[15] Joshi S, Davies H, Sims LP, Levy SE, Dean J (2007) Ovarian gene expression in the absence of FIGLA, an oocyte-specific transcription factor. BMC Dev Biol 7: 67 17567914 10.1186/1471-213X-7-67 PMC1906760

[16] Hamatani T, Falco G, Carter MG, Akutsu H, Stagg CA, et al (2004) Age-associated alteration of gene expression patterns in mouse oocytes. Hum Mol Genet 13: 2263–2278. 15317747 10.1093/hmg/ddh241

[17] Li L, Baibakov B, Dean J (2008) A subcortical maternal complex essential for preimplantation mouse embryogenesis. Dev Cell 15: 416–425. 10.1016/j.devcel.2008.07.010 18804437 PMC2597058

[18] Kim KH, Lee KA (2014) Maternal effect genes: Findings and effects on mouse embryo development. Clin Exp Reprod Med 41: 47–61. 10.5653/cerm.2014.41.2.47 25045628 PMC4102690

[19] Zernicka-Goetz M (1994) Activation of embryonic genes during preimplantation rat development. Mol Reprod Dev 38: 30–35. 8049062 10.1002/mrd.1080380106

[20] Schultz RM (1993) Regulation of zygotic gene activation in the mouse. Bioessays 15: 531–538. 8135766 10.1002/bies.950150806

[21] Zeng F, Baldwin DA, Schultz RM (2004) Transcript profiling during preimplantation mouse development. Dev Biol 272: 483–496. 15282163 10.1016/j.ydbio.2004.05.018

[22] Hamatani T, Carter MG, Sharov AA, Ko MS (2004) Dynamics of global gene expression changes during mouse preimplantation development. Dev Cell 6: 117–131. 14723852 10.1016/s1534-5807(03)00373-3

[23] Wang QT, Piotrowska K, Ciemerych MA, Milenkovic L, Scott MP, et al (2004) A genome-wide study of gene activity reveals developmental signaling pathways in the preimplantation mouse embryo. Dev Cell 6: 133–144. 14723853 10.1016/s1534-5807(03)00404-0

[24] Paynton BV, Rempel R, Bachvarova R (1988) Changes in state of adenylation and time course of degradation of maternal mRNAs during oocyte maturation and early embryonic development in the mouse. Dev Biol 129: 304–314. 2458285 10.1016/0012-1606(88)90377-6

[25] Alizadeh Z, Kageyama S, Aoki F (2005) Degradation of maternal mRNA in mouse embryos: selective degradation of specific mRNAs after fertilization. Mol Reprod Dev 72: 281–290. 16094646 10.1002/mrd.20340

[26] Lee HS, Kim EY, Lee KA (2011) Changes in gene expression associated with oocyte meiosis after Obox4 RNAi. Clin Exp Reprod Med 38: 68–74. 10.5653/cerm.2011.38.2.68 22384421 PMC3283059

[27] Park GT, Lee KA (2013) Nuclear localization of Obox4 is dependent on its homeobox domain. Clin Exp Reprod Med 40: 1–6. 10.5653/cerm.2013.40.1.1 23614109 PMC3630287

[28] Ohsugi M, Zheng P, Baibakov B, Li L, Dean J (2008) Maternally derived FILIA-MATER complex localizes asymmetrically in cleavage-stage mouse embryos. Development 135: 259–269. 18057100 10.1242/dev.011445

[29] Tadros W, Lipshitz HD (2009) The maternal-to-zygotic transition: a play in two acts. Development 136: 3033–3042. 10.1242/dev.033183 19700615

[30] Tsai TC, Lin W, Yang SH, Cheng WT, Cheng EH, et al (2010) Granzyme G is expressed in the two-cell stage mouse embryo and is required for the maternal-zygotic transition. BMC Dev Biol 10: 88 10.1186/1471-213X-10-88 20704734 PMC2930601

[31] Rother F, Shmidt T, Popova E, Krivokharchenko A, Hugel S, et al (2011) Importin alpha7 is essential for zygotic genome activation and early mouse development. PLoS One 6: e18310 10.1371/journal.pone.0018310 21479251 PMC3066239

[32] Golbus MS, Calarco PG, Epstein CJ (1973) The effects of inhibitors of RNA synthesis (alpha-amanitin and actinomycin D) on preimplantation mouse embryogenesis. J Exp Zool 186: 207–216. 4795793 10.1002/jez.1401860211

[33] Qiu JJ, Zhang WW, Wu ZL, Wang YH, Qian M, et al (2003) Delay of ZGA initiation occurred in 2-cell blocked mouse embryos. Cell Res 13: 179–185. 12862318 10.1038/sj.cr.7290162

[34] Lykke-Andersen K, Gilchrist MJ, Grabarek JB, Das P, Miska E, et al (2008) Maternal Argonaute 2 is essential for early mouse development at the maternal-zygotic transition. Mol Biol Cell 19: 4383–4392. 10.1091/mbc.E08-02-0219 18701707 PMC2555945

[35] Ma J, Zeng F, Schultz RM, Tseng H (2006) Basonuclin: a novel mammalian maternal-effect gene. Development 133: 2053–2062. 16624857 10.1242/dev.02371

[36] Wan LB, Pan H, Hannenhalli S, Cheng Y, Ma J, et al (2008) Maternal depletion of CTCF reveals multiple functions during oocyte and preimplantation embryo development. Development 135: 2729–2738. 10.1242/dev.024539 18614575 PMC2596970

[37] Yurttas P, Vitale AM, Fitzhenry RJ, Cohen-Gould L, Wu W, et al (2008) Role for PADI6 and the cytoplasmic lattices in ribosomal storage in oocytes and translational control in the early mouse embryo. Development 135: 2627–2636. 10.1242/dev.016329 18599511 PMC2708103

[38] Kim KH, Kim EY, Kim Y, Kim E, Lee HS, et al (2011) Gas6 downregulation impaired cytoplasmic maturation and pronuclear formation independent to the MPF activity. PLoS One 6: e23304 10.1371/journal.pone.0023304 21850267 PMC3151302

